# The Role of Stroke as a Trigger for Incident Venous Thromboembolism: Results from a Population-based Case-Crossover Study

**DOI:** 10.1055/s-0039-1681020

**Published:** 2019-02-22

**Authors:** Vânia M. Morelli, Joakim K. Sejrup, Birgit Småbrekke, Ludvig B. Rinde, Gro Grimnes, Trond Isaksen, John-Bjarne Hansen, Kristian Hindberg, Sigrid K. Brækkan

**Affiliations:** 1 K. G. Jebsen Thrombosis Research and Expertise Center (TREC), Department of Clinical Medicine, UiT - The Arctic University of Norway, Tromsø, Norway; 2 Division of Internal Medicine, University Hospital of North Norway, Tromsø, Norway

**Keywords:** venous thromboembolism, stroke, risk factor, immobilization, infection

## Abstract

Stroke is associated with a short-term increased risk of subsequent venous thromboembolism (VTE). It is unclear to what extent this association is mediated by stroke-related complications that are potential triggers for VTE, such as immobilization and infection. We aimed to investigate the role of acute stroke as a trigger for incident VTE while taking other concomitant VTE triggers into account. We conducted a population-based case-crossover study with 707 VTE patients. Triggers were registered during the 90 days before a VTE event (hazard period) and in four preceding 90-day control periods. Conditional logistic regression was used to estimate odds ratios with 95% confidence intervals (CIs) for VTE according to triggers. Stroke was registered in 30 of the 707 (4.2%) hazard periods and in 6 of the 2,828 (0.2%) control periods, resulting in a high risk of VTE, with odds ratios of 20.0 (95% CI: 8.3–48.1). After adjustments for immobilization and infection, odds ratios for VTE conferred by stroke were attenuated to 6.0 (95% CI: 1.6–22.1), and further to 4.0 (95% CI: 1.1–14.2) when other triggers (major surgery, red blood cell transfusion, trauma, and central venous catheter) were added to the regression model. A mediation analysis revealed that 67.8% of the total effect of stroke on VTE risk could be mediated through immobilization and infection. Analyses restricted to ischemic stroke yielded similar results. In conclusion, acute stroke was a trigger for VTE, and the association between stroke and VTE risk appeared to be largely mediated by immobilization and infection.

## Introduction

Stroke is a major cause of death and disability worldwide. 1 Furthermore, patients with acute stroke are at risk of developing venous thromboembolism (VTE). 2 3 4 5 In a meta-analysis involving patients with acute ischemic stroke from several randomized controlled trials, the incidence of VTE among stroke patients who did not receive antithrombotic therapy during follow-up was 17% for asymptomatic and symptomatic deep vein thrombosis (DVT). 3 Although clinically overt pulmonary embolism (PE) occurs in only 1% of patients during the first 14 days after an acute stroke, 2 3 6 PE may account for up to 25 to 50% of deaths after acute stroke. 2 7 8 In population-based studies, the risk of VTE has been shown to be particularly high during the first three months after stroke. 4 5 For instance, data from the Tromsø Study revealed a 20- and 11-fold increased risk of VTE in the first and subsequent two months after ischemic stroke, respectively, with a rapid decline of the risk estimates thereafter. 5

Even though the association between stroke and subsequent risk of VTE is well established, 2 3 4 5 the mechanism underlying this association is not fully understood, as data on explanatory factors are scarce. Confounding due to the presence of common atherosclerotic risk factors seems to poorly explain this association, since among the atherosclerotic risk factors, only advancing age and obesity have consistently been associated with VTE. 9 If causal, the association between stroke (exposure) and VTE (outcome) may be explained by factors that are a consequence of stroke (intermediates or mediators), which in turn could increase the risk of VTE. 10 Such an effect of the exposure on the outcome is indirect as it acts through the intermediate variable, whereas the effect that is not explained by the intermediate is referred to as a direct effect. 10

Neurological and medical complications can frequently arise as consequences of acute stroke, and lead to prolonged hospitalization, poorer functional outcome, and increased mortality rate of stroke patients. 11 12 13 Neurological deficits entailing immobilization and infections are common complications after stroke 11 13 that have the potential to trigger a VTE event, 14 15 16 and could therefore be intermediates in the chain of causation between stroke and VTE. Indeed, the short-term increased risk of VTE after acute stroke 5 suggests that stroke-related complications are main contributors to the VTE risk in stroke patients. In the relationship between stroke and VTE, infection can also act as confounder, particularly pneumonia that has been reported to be associated with increased risk of both ischemic stroke and VTE. 17 Additionally, pneumonia is one of the most frequent medical complications following acute stroke, 12 13 and multiple factors have been shown to independently contribute to stroke-related pneumonia, including dysphagia with aspiration of oropharyngeal material, advancing age, and severity of poststroke disability. 18 19 Enhanced knowledge on how much immobilization and infection mediate the association between stroke and risk of VTE is clinically relevant, since it may provide opportunity for targeted interventions to improve prevention of VTE after stroke.

In this study, we aimed to assess the role of acute stroke as a trigger for VTE while taking other concomitant VTE triggers into account, and to investigate to what extent immobilization and infection could mediate the effect of stroke on VTE risk. For this purpose, we conducted a case-crossover study with incident VTE cases recruited from the general population. This study design relies on intra-person comparison, since each case serves as his or her own control, and it is suited to investigate the effects of transient exposures on acute outcomes. 20

## Methods

### Study Population

Participants were recruited from the fourth survey of the Tromsø Study, a single-center, population-based cohort study, details of which have been described elsewhere. 21 Briefly, in 1994 to 1995, all inhabitants aged >24 years living in the municipality of Tromsø were invited, and 27,158 (77% of the eligible population) participated. Incident VTE events among the study participants were recorded from the date of enrollment (1994–1995) until December 31, 2012. 22 All incident VTE events were identified by searching the hospital discharge diagnosis registry, the autopsy registry, and the radiology procedure registry at the University Hospital of North Norway. The University Hospital of North Norway is the only hospital in the region, and all hospital care and relevant diagnostic radiology is provided exclusively by this hospital. The medical record for each potential case of VTE was reviewed by trained personnel, and an episode of VTE was confirmed and registered as a validated VTE when clinical signs and symptoms of DVT or PE were combined with objective confirmation by radiological procedures, and resulted in a VTE diagnosis requiring treatment, as described in detail previously. 22 The study was approved by the regional committee for research ethics, and all participants gave their informed written consent to participate.

### Study Design

**Fig. 1:**
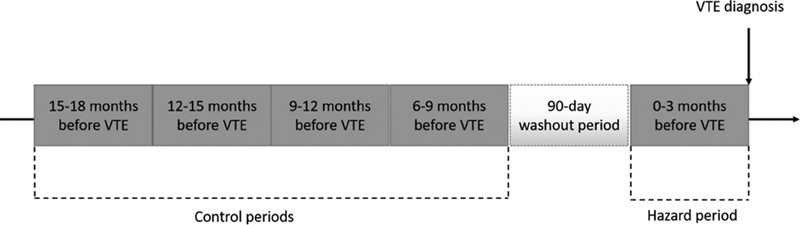
Case-crossover study design. A transient risk factor, or trigger, was recorded for each case of venous thromboembolism (VTE) in the 90-day hazard period prior to the event, and in four preceding 90-day control periods, separated by a 90-day washout period.

A case-crossover study was conducted to investigate the role of acute stroke as a trigger for VTE. This design uses data on cases only, that is, on individuals who have experienced the outcome of interest. 20 In most observational study designs, confounding remains a methodological challenge. In the case-crossover study, individuals serve as their own controls, and all potential fixed confounders, such as chronic conditions, comorbidities, and anthropometric and genetic factors, are largely controlled for through the study design. 20 As previously described, 16 the study population comprised all incident VTE cases ( *n* = 707) that occurred among the participants of the Tromsø Study during 1994 to 2012. In this study, the hazard (i.e., risk) period was defined as the 90-day period before the date of the incident VTE. 15 16 Exposures during the hazard period were compared with exposures occurring during the four previous 90-day control periods ( Fig. 1 ). The length of these hazard and control periods was predefined based on the definition of provoking factors, as described by Kearon et al. 23 A 90-day washout period between the control and the hazard periods was included to avoid carry-over effects. For each VTE case, trained medical personnel searched the hospital medical records for relevant risk factors, diagnostic procedures, surgical and medical treatment, laboratory tests and diagnoses during hospital admissions, day care, and outpatient clinic visits in any of the control or hazard periods. We did not have access to medical records from general practice. 16

### Definition of Transient Risk Factors for VTE

A transient risk factor, or trigger, was defined by its presence during the 90 days before a VTE event (hazard period) and/or in four preceding 90-day control periods. 16 If an exposure occurred over several days, it was considered to have occurred if any of the days of the exposure fell within the specified 90-day time period. Stroke was defined in the presence of the diagnosis of ischemic, hemorrhagic, or unclassified stroke in the medical records. The other VTE triggers were recorded as previously described. 16 Briefly, immobilization was defined by the presence of bed rest for 3 days or more, ECOG (Eastern Cooperative Oncology Group) score of 4, or other immobilizing factors specified in the patient's medical record (e.g., confinement to wheelchair). Infection was recorded if an acute infection was noted by a physician in the patient's medical record, and this definition included both community-acquired infections that required hospital admission and hospital-acquired infections. Infection was defined as respiratory tract infection (RTI), urinary tract infection, and other infections. As RTI and PE may have similar symptoms, some PEs could initially have been diagnosed as RTI. Therefore, all cases with RTI and PE were thoroughly reevaluated by a specialist in infectious diseases, and the diagnoses of RTI that were most likely incorrect ( *n* = 8) were recoded as “no RTI.” Red blood cell transfusion, central venous catheterization, trauma, and major surgery were recorded if noted in the medical record.

### Statistical Analyses

Statistical analyses were performed using STATA version 15.0 (Stata Corporation, College Station, Texas, United States). We used conditional logistic regression to calculate odds ratios (ORs) with 95% confidence intervals (CIs) as estimates of the relative risk of VTE according to the presence of stroke, immobilization, and acute infection, with the reference category being defined as no exposure to the aforementioned variables. In model 1, we calculated the crude association between stroke and VTE. Model 2 was adjusted for the presence of immobilization and infection. In model 3, we additionally adjusted for major surgery, red blood cell transfusion, trauma, and central venous catheter, since these variables are potential triggers for VTE that often coexist with both infection and immobilization. 15 16 Risk estimates for VTE associated with immobilization and infection were also presented, using the same models. However, for immobilization, models were adjusted for stroke and infection, and for infection, models were adjusted for stroke and immobilization.

Under the assumption that immobilization and infection were a consequence of acute stroke, we further examined to what extent both factors could mediate the association between stroke and VTE using the method developed by Karlson, Holm, and Breen (KHB method). 24 The technical details and mathematical proofs of this method are available elsewhere. 24 Briefly, this method estimates all effects (i.e., direct, indirect, and total) on the same scale and the coefficients in conditional logistic regression models are not affected by rescaling, particularly when the total effect is decomposed into the direct and indirect effects. This property allows us to compare the coefficients without any scale identification issues. Additionally, the KHB method has the important feature that it can handle more than one mediator simultaneously. This method also enables to decompose the contribution from the different mediators while adjusting for other factors.

In addition to the overall analyses, we performed subgroup analyses stratified by the localization of the thrombotic event, i.e., DVT and PE with or without DVT. We repeated all analyses considering only ischemic stroke as an exposure. For sensitivity purposes, we also conducted analyses for overall VTE where subjects with active cancer at the time of VTE diagnosis were excluded. To account for seasonality of infection in the association between acute infection and risk of VTE, we performed a sensitivity analysis restricting the comparison of the hazard period with the control period that occurred 12 to 15 months before VTE. According to our study design ( Fig. 1 ), this particular control period for each study participant represents exactly the same calendar period 1 year prior to the VTE event (i.e., the exactly same season as the hazard period).

## Results

Table 1 shows the characteristics of the study participants at the time of VTE, and the distribution of VTE triggers in the hazard and control periods. Median age at VTE was 71 years, and 53.6% were women. Among the VTE events, 57.7% were DVT and 42.3% were PE with or without DVT. In 19.1% of the cases, VTE occurred during hospitalization for other conditions. All potential triggers for VTE, including stroke, occurred more frequently in the hazard than control periods. Stroke occurred in 30 of the 707 (4.2%) hazard periods, and in 6 of the 2,828 (0.2%) control periods. Among the stroke events ( *n* = 36), 23 were recorded as ischemic (20 occurred in the hazard and 3 in the control periods), 9 as hemorrhagic (7 occurred in the hazard and 2 in the control periods), and 4 as unclassified (3 occurred in the hazard and 1 in the control periods). Among the 30 cases with stroke in the hazard period, 24 (80%) developed their VTE during the hospitalization with stroke, whereas 6 (20%) developed their VTE in the community after being discharged with stroke. Thromboprophylaxis with low-molecular weight heparin (LMWH) was prescribed in 138 of the 707 (19.5%) hazard periods, and in 78 of the 2,828 (2.8%) control periods. LMWH was prescribed in 13 of the 30 (43.3%) patients with stroke in the hazard period, and in none of the 6 patients with stroke in the control periods.

**Table 1:** Characteristics of the study participants

Characteristics	At time of VTE diagnosis ( *n* = 707)	
Median age, years ± SD	71 ± 14	
Female sex ( *n* , %)	379 (53.6)	
Deep vein thrombosis ( *n* , %)	408 (57.7)	
Pulmonary embolism a ( *n* , %)	299 (42.3)	
VTE during hospitalization ( *n* , %)	135 (19.1)	
**Triggers of VTE**	**Hazard period** **(** ***n*** ** = 707)**	**Control period** **(** ***n*** ** = 2,828)**
Stroke b ( *n* , %)	30 (4.2)	6 (0.2)
Immobilization c ( *n* , %)	222 (31.4)	57 (2.0)
Infection ( *n* , %)	267 (37.8)	107 (3.8)
Major surgery ( *n* , %)	118 (16.7)	88 (3.1)
Red blood cell transfusion ( *n* , %)	82 (11.6)	28 (1.0)
Trauma ( *n* , %)	71 (10.0)	25 (0.9)
Central venous catheter ( *n* , %)	56 (7.9)	17 (0.6)

Abbreviation: SD, standard deviation; VTE, venous thromboembolism.

a Pulmonary embolism with or without deep vein thrombosis.

b Ischemic, hemorrhagic, or unclassified stroke.

c Bed rest for ≥3 days, Eastern Cooperative Oncology Group score of 4, or other immobilizing factors specified in the patient's medical record (e.g., confinement to wheelchair).

Frequencies of stroke, immobilization, and infection during the hazard and control periods, as well as risk estimates for VTE associated with each variable, are described in Table 2 . In unadjusted models, stroke, immobilization, and infection were all associated with an increased risk of VTE. The risk of VTE conferred by stroke was high, with an OR of 20 (95% CI: 8.3–48.1). However, after adjustment for immobilization and infection (model 2), the association between stroke and VTE was markedly attenuated, resulting in an OR for VTE of 6.0 (95% CI: 1.6–22.1). Of note, when immobilization and infection were added separately to the regression models, the effect of stroke on the risk of VTE was attenuated to a similar extent when adjusted for immobilization only (OR: 11.5, 95% CI: 3.2–41.0) or infection only (OR: 9.0, 95% CI: 3.1–26.0). When the other VTE triggers (i.e., major surgery, trauma, red blood cell transfusion, and central venous catheter) were introduced in a third model along with immobilization and infection, risk estimates for VTE associated with stroke were further attenuated to 4.0 (95% CI: 1.1–14.2).

**Table 2:** Distribution of triggers in the hazard and control periods and odds ratios (ORs) of venous thromboembolism

	Hazard period ( *n* = 707) *n* (%)	Control periods ( *n* = 2,828) a *n* (%)	Model 1 OR (95% CI)	Model 2 OR (95% CI)	Model 3 OR (95% CI)
Stroke	30 (4.2)	6 (0.2)	20.0 (8.3–48.1)	6.0 (1.6–22.1)	4.0 (1.1–14.2)
Immobilization	222 (31.4)	57 (2.0)	66.7 (37.3–119.4)	37.0 (19.9–69.0)	26.5 (14.0–50.1)
Infection	267 (37.8)	107 (3.8)	24.2 (17.2–34.0)	14.3 (9.9–20.9)	11.7 (7.9- 17.2)

Abbreviations: CI, confidence interval.

Note: For immobilization, infection, and stroke, the reference category was defined as no exposure to the trigger.

Model 1: unadjusted odds ratios.

Model 2: adjusted for the other variables in this table.

Model 3: adjusted as in model 2 with addition of major surgery, trauma, red blood cell transfusion, and central venous catheter.

a 707 cases, four control periods for each case.

Next, we analyzed the magnitude of the mediating effect of immobilization and infection on the relationship between stroke and VTE. In a mediation analysis adjusted for major surgery, trauma, red blood cell transfusion, and central venous catheter, about two-thirds (67.8%) of the total effect of stroke on VTE risk was due to a mediating effect (i.e., indirect effect) acting through immobilization and infection ( Supplementary Table S1 ). With respect to the mediating effect, 56.7% was attributable to immobilization and 43.3% to infection.

Table 3 shows frequencies and ORs for stroke, immobilization, and infection in subjects with DVT and PE separately. In unadjusted models, the thrombosis risk conferred by stroke was higher for DVT (OR: 24.0, 95% CI: 7.1–81.5) than for PE (OR: 16.0, 95% CI: 4.5–56.7). Still, the association between stroke and DVT or PE was again substantially attenuated after adjustments for all VTE triggers (model 3), with ORs of 4.0 (95% CI: 0.6–27.9) and 4.1 (95% CI: 0.7–23.0), respectively.

**Table 3:** Distribution of triggers in the hazard and control periods and odds ratios (ORs) of deep vein thrombosis and pulmonary embolism

Deep vein thrombosis	Hazard period ( *n* = 408) *n* (%)	Control periods ( *n* = 1,632) a *n* (%)	Model 1 OR (95% CI)	Model 2 OR (95% CI)	Model 3 OR (95% CI)
Stroke	18 (4.4)	3 (0.2)	24.0 (7.1–81.5)	8.6 (1.3–57.3)	4.0 (0.6–27.9)
Immobilization	143 (35.0)	38 (2.3)	73.6 (34.4–157.4)	40.5 (17.9–91.6)	29.9 (13.1–68.2)
Infection	143 (35.0)	60 (3.7)	19.9 (13.0–30.6)	8.8 (5.4–14.3)	6.9 (4.1–11.6)
**Pulmonary embolism**	**Hazard period** **(** ***n*** ** = 299)** ***n*** **(%)**	**Control periods** **(** ***n*** ** = 1196)** a ***n*** **(%)**	**Model 1** **OR (95% CI)**	**Model 2** **OR (95% CI)**	**Model 3** **OR (95% CI)**
Stroke	12 (4.0)	3 (0.3)	16.0 (4.5–56.7)	4.8 (0.8–27.3)	4.1 (0.7–23.0)
Immobilization	79 (26.4)	19 (1.6)	57.0 (23.0–141.0)	36.5 (13.5–98.4)	27.0 (9.7–75.3)
Infection	124 (41.5)	47 (3.9)	32.4 (18.2–57.5)	25.8 (13.8–48.0)	21.5 (11.4–40.5)

Abbreviations: CI, confidence interval.

Note: For immobilization, infection, and stroke, the reference category was defined as no exposure to the trigger.

Model 1: unadjusted odds ratios.

Model 2: adjusted for the other variables in this table.

Model 3: adjusted as in model 2 with addition of major surgery, trauma, red blood cell transfusion, and central venous catheter.

a 408 deep vein thrombosis cases, four control periods for each case; 299 pulmonary embolism cases, four control periods for each case.

When the analysis was restricted to subjects exposed to ischemic stroke, the results were similar to those obtained when all types of stroke were taken into account, for overall VTE, DVT and PE, and in mediation analysis for overall VTE ( Supplementary Tables S2–S4 ). Ischemic stroke was associated with an OR for VTE of 26.7 (95% CI: 7.9–89.7) in an unadjusted model ( Supplementary Table S2 ). However, after adjustment for immobilization and infection (model 2) or for all VTE triggers (model 3), ORs for VTE conferred by ischemic stroke were attenuated to 4.9 (95% CI: 0.8–29.4) and 3.7 (95% CI: 0.7–18.7), respectively. Among the 707 cases, 176 (24.9%) had active cancer at the time of VTE diagnosis. Exclusion of these patients yielded similar results to the main analysis ( Supplementary Table S5 ). When the hazard period was compared with the control period that occurred 12 to 15 months before VTE (i.e., the control period that represented the same seasons as the hazard period), the OR for VTE associated with infection remained high (OR: 18.3, 95% CI: 10.7–31.3) and did not significantly differ from the overall OR where all control periods were taken into account (OR: 24.2, 95% CI: 17.2–34.0; Table 2 ).

## Discussion

In this population-based case-crossover study of 707 patients with incident VTE, we investigated the role of acute stroke as a VTE trigger, and found that stroke was associated with a substantial increased risk of subsequent VTE, with an OR of 20.0. However, the impact of stroke on the risk of VTE was largely attenuated after adjustments for other VTE triggers, mainly immobilization and infection, which are both common stroke-related complications. Indeed, almost 68% of the total effect of stroke on the risk of VTE was indirect, due to the mediating effect of immobilization and infection. It is noteworthy that even after adjustments for all the potential VTE triggers (i.e., immobilization, infection, surgery, red blood cell transfusion, trauma, and central venous catheter), the risk of VTE associated with stroke remained elevated, with an OR of 4.0. Analyses restricted to subgroups (i.e., DVT and PE) or ischemic stroke yielded similar results. Our findings suggest that immobilization and infection are important intermediates for the association between stroke and subsequent risk of VTE, but conditions related to stroke other than these intermediates studied could still play a considerable role in the risk of VTE.

Studies aimed at investigating the mechanism underlying the association between stroke and subsequent risk of VTE have been scarce thus far. Recently, we have demonstrated in the Tromsø Study 5 that shared atherosclerotic risk factors are an unlikely explanation for the association between stroke and VTE due to the short-term risk of VTE after ischemic stroke, and to the marginal impact that adjustments for atherosclerotic risk factors had on this association. Findings from the present study further suggest that immobilization and infection are main explanatory factors for the association between stroke and VTE, accounting for about two-thirds of the total effect of stroke on the risk of VTE. To the best of our knowledge, this is the first study that has evaluated the role of stroke as a VTE trigger using a case-crossover design. Of note, our results are consistent with previous reports in which stroke-related factors, such as severity of neurological impairment 25 and lower limb paresis, 26 were significant contributors to the VTE risk in stroke patients.

Immobilization and infection may contribute to the increased risk of VTE after stroke either via prolongation of hospital stay or by themselves, probably with the involvement of multiple coexisting pathways. Immobilization is an important risk factor for VTE 14 due to venous stasis. Stroke patients are often temporarily immobilized owing to bed rest or neurological deficits of affected limbs, 26 27 and thus more prone to venous thrombus formation. Infections, particularly urinary tract infection and pneumonia, are leading medical complications after stroke, 12 and stroke severity is an independent predictor of both urinary tract infection 28 and pneumonia. 29 In a meta-analysis involving 137,817 stroke patients, the overall rate of infection in the acute phase of stroke was 30%, and among those admitted to an intensive care unit, rates were as high as 45%. 13 Infection can increase the risk of VTE either through a systemic activation of the coagulation system 17 or through immobilization/bed rest. 30 Potential mechanisms related to the coagulation system underlying the link between infection and VTE include upregulation of tissue factor, a main trigger of blood coagulation in vivo, and downregulation of anticoagulant factors (e.g., activated protein C). 17 Infection is also associated with the formation of neutrophil extracellular traps (NETs), which are produced to allow neutrophils to trap and disarm microbes in the extracellular environment. 31 NETs provide a new link between innate immunity and thrombosis and have been shown to contribute to experimental DVT. 31 Cell-free DNA is a key component of NETs that may exert harmful effects by triggering blood coagulation via the contact pathway, in a FXII- and FXI-dependent manner. 32 Finally, the relationship between infection and immobilization is bidirectional, as immobilization is a risk factor for infection, particularly pneumonia, 33 and their combination has been suggested to have synergistic effects on VTE risk. 16

It is of interest that the risk of VTE conferred by stroke, albeit attenuated, remained considerably elevated even after adjustment for immobilization and infection (OR: 6.0), and further for the other VTE triggers (OR: 4.0). In a case-crossover study, all fixed confounders that do not vary over the study periods are largely controlled for through the design, 20 and are therefore unlikely to influence our results. The association between stroke and VTE that nevertheless persisted after multivariable adjustment could be due to other unknown or unmeasured transient factors related to stroke itself that had the potential to increase the risk of VTE. For instance, previous studies have shown that levels of several biomarkers of coagulation activation were increased after acute stroke and associated with stroke severity. 34 35 A hypercoagulable state is a key pathway for venous thrombus formation, 36 and could have accounted for part of the observed effect of acute stroke on VTE risk. However, our study was not designed to evaluate the validity of this proposed mechanism, and it remains to be clarified to what extent a hypercoagulable state poststroke may contribute to the subsequent risk of VTE.

Our findings may have some clinical implications. Given the potential role of infection and immobilization in mediating the relationship between stroke and VTE, their prevention through improvement in patient care and rehabilitation programs may lower the risk of VTE after acute stroke. For instance, the addition of a passive turning and mobilization program to usual care effectively reduced the incidence of pneumonia during the acute phase of stroke. 33 Our findings also suggest the need to consider not only immobilization but also infection in clinical decision making regarding thromboprophylaxis after stroke. For prevention of VTE in patients with acute ischemic stroke and restricted mobility, current guidelines recommend the use of prophylactic doses of LMWH or unfractionated heparin, which should be initiated as early as possible, and continued throughout the hospital stay or until the patient has regained mobility. 37 A meta-analysis of randomized clinical trials implied that prophylaxis with LMWH or unfractionated heparin in patients with acute ischemic stroke had the potential to reduce the incidence of symptomatic DVT by 70% and the incidence of fatal and nonfatal PE by 30%. 37 Still, data from real-world practice have shown that less than half of the patients hospitalized for ischemic stroke at risk of VTE receive any form of thromboprophylaxis. 38 39 Uncertainty on the identification of high risk groups for VTE, and the perceived bleeding risk associated with anticoagulation, may explain, at least in part, the current underuse of thromboprophylaxis after acute stroke. Indeed, decisions on thromboprophylaxis and its duration after acute stroke could be a dilemma in the clinics, as the benefits of prophylaxis with LMWH in reducing the risk of VTE may be offset by major bleeding, 40 41 including intracerebral hemorrhage. 40 In the randomized controlled EXCLAIM trial, which involved 389 patients with acute ischemic stroke, extended-duration prophylaxis with LMHW was associated with a reduction in the incidence of VTE but also with an increase in major bleeding. 41 An accurate prediction of VTE after stroke may guide clinical decisions, and thereby increase the use of anticoagulants in patients with a favorable benefit-to-harm ratio for thromboprophylaxis. However, data on prediction of VTE in stroke patients are scarce. Currently existing risk prediction algorithms for VTE discriminated poorly between immobile stroke patients at high and low risk of VTE, 42 or involved a limited number of patients, who were all Asians. 43 44 Taken together, our results suggest that infection plays an important role in the development of VTE after acute stroke, a finding that requires confirmation by future investigation in a cohort of stroke patients. The addition of infection to future prediction models may improve the identification of stroke patients at a substantially high risk of developing VTE, who would benefit most from thromboprophylaxis with anticoagulation.

The strengths of our study include the high attendance rate in the population-based cohort where the cases were recruited from, the complete and validated registry of VTE events, and the study design enabling us to focus on transient risk factors while controlling for potential fixed confounders, as participants serve as their own controls. 20 Some limitations should be addressed as well. First, in this study, information on exposure to VTE triggers was obtained during the last 90 days before each admission, but without dissecting the temporal sequence between them. Analyses of the present study assume that stroke preceded the occurrence of immobilization, infection, and the other VTE triggers studied. However, although it is unlikely, we cannot rule out that acute stroke, in some cases, may have followed the other VTE triggers during hospitalization. Therefore, our findings should be interpreted with caution, and they need to be confirmed by future prospective cohort studies involving stroke patients, in which a clear temporal sequence between exposure, intermediate, and outcome is determined through the design. Second, since exposure to stroke was based on data obtained from the review of medical records, with no further validation by an independent end-point committee, misclassification cannot be ruled out. Nevertheless, the definition of stroke is currently based on objective diagnostic criteria, in which radiological procedures play a central role, 45 thereby making misclassification of stroke diagnosis unlikely. Third, information on exposures in our case-crossover study might be subject to bias, as doctors could be more aware of VTE-risk factors when VTE is suspected than during admissions for other conditions in the control periods. For instance, as immobilization is a well-known risk factor for VTE, immobilization could have been more recorded when VTE was suspected. If so, the effect of immobilization on the risk of VTE and its role as a mediator for the association between stroke and VTE might be overestimated. In this study, we only had access to medical records from hospital, and therefore less severe infections and conditions leading to immobilization managed solely in general practice were not included in our analysis. A higher number of underreported exposure to immobilization and infection (due to management by general practice) in the control than hazard periods could also have led to an overestimation of the impact of these exposures on VTE risk. Taken together, potential misclassification of immobilization and infection during the hazard and control periods might have limited the internal validity of our results. Fourth, even if fixed confounders are controlled for through the study design, other (unknown/unmeasured) transient risk factors might have influenced the relationship between stroke and VTE, as we have previously pointed out. Fifth, unfortunately, we did not have information about subtypes of ischemic stroke, stroke severity, and medications prescribed according to stroke subtype, such as the use of anticoagulants among patients with cardioembolic stroke. The aforementioned information would allow a more detailed assessment of the role of stroke as a VTE trigger. Sixth, the present findings should be interpreted with caution, as low numbers of exposure to stroke limited the statistical power of our results in some subgroups, particularly when assessing the role of ischemic stroke as a VTE trigger.

In conclusion, acute stroke was a trigger for VTE in this case-crossover study, but our findings suggest that the association between acute stroke and subsequent risk of VTE is largely mediated by the presence of immobilization and infection.
