# Supplementary material for: The Role of Stroke as a Trigger for Incident Venous Thromboembolism: Results from a Population-based Case-Crossover Study
Source: TH Open. 2019 Feb 22;3(1):e50–7. doi: 10.1055/s-0039-1681020 (PMC6524907; doi:10.1055/s-0039-1681020)
Supplement: Supplementary file 1 — Supplementary Material [file 10-1055-s-0039-1681020-s180053.pdf]

Supplementary Table S1 The KHB mediation analysis and decomposition results for the association between stroke and venous thromboembolism

Conditional logistic regression			
	**Coefficient**	**SE**	**Mediation percentage**
Coefficients			
Total effect	4.29	0.68	–
Direct effect	1.38	0.65	–
Mediating effect	2.91[a]	0.68	67.8
Through			
Infection	1.26	0.22	43.3
Immobilization	1.65	0.34	56.7

Abbreviation: SE, standard error.
Note: The Karlson–Holm–Breen (KHB) method was used to perform the mediation analysis.[1,2]
[a]p-Value < 0.005.

Supplementary Table S2 Distribution of triggers in the hazard and control periods and odds ratios (ORs) of venous thromboembolism

	**Hazard period ($n = 707$) n (%)**	**Control periods ($n = 2{,}828$)[a] n (%)**	**Model 1 OR (95% CI)**	**Model 2 OR (95% CI)**	**Model 3 OR (95% CI)**
Ischemic stroke	20 (2.8)	3 (0.1)	26.7 (7.9–89.7)	4.9 (0.8–29.4)	3.7 (0.7–18.7)
Immobilization	222 (31.4)	57 (2.0)	66.7 (37.3–119.4)	37.5 (20.2–69.6)	27.2 (14.4–51.2)
Infection	267 (37.8)	107 (3.8)	24.2 (17.2–34.0)	14.4 (9.9–20.9)	11.7 (8.0–17.3)

Abbreviations: CI, confidence interval.
Note: For immobilization, infection and ischemic stroke, the reference category was defined as no exposure to the trigger.
Model 1: unadjusted odds ratios.
Model 2: adjusted for the other variables in this table.
Model 3: adjusted as in model 2 with addition of major surgery, trauma, red blood cell transfusion, and central venous catheter.
[a]707 cases, four control periods for each case.

Supplementary Table S3 The KHB mediation analysis and decomposition results for the association between ischemic stroke and venous thromboembolism

Conditional logistic regression			
	**Coefficient**	**SE**	**Mediation percentage**
Coefficients			
Total effect	4.69	0.86	–
Direct effect	1.30	0.83	–
Mediating effect	3.39[a]	0.76	72.3
Through			
Infection	1.53	0.25	45.1
Immobilization	1.86	0.41	54.9

Abbreviation: SE, standard error.
Note: Karlson–Holm–Breen (KHB) method was used to perform the mediation analysis.[1,2]
[a]p-Value < 0.005.

Supplementary Table S4 Distribution of triggers in the hazard and control periods and odds ratios (ORs) of deep vein thrombosis and pulmonary embolism

Deep vein thrombosis	Hazard period ($n = 408$) n (%)	Control periods ($n = 1632$)[a] n (%)	Model 1 OR (95% CI)	Model 2 OR (95% CI)	Model 3 OR (95% CI)
Ischemic stroke	13 (3.2)	2 (0.1)	26.0 (5.9–115.2)	8.7 (1.0–77.2)	4.1 (0.5–34.7)
Immobilization	143 (35.0)	38 (2.3)	73.6 (34.4–157.4)	40.4 (18.0–91.1)	30.3 (13.3–69.0)
Infection	143 (35.0)	60 (3.7)	19.9 (13.0–30.6)	8.6 (5.5–14.4)	7.0 (4.2–11.7)
**Pulmonary embolism**	**Hazard period ($n = 299$) n (%)**	**Control periods ($n = 1196$)[a] n (%)**	**Model 1 OR (95% CI)**	**Model 2 OR (95% CI)**	**Model 3 OR (95% CI)**
Ischemic stroke	7 (2.3)	1 (0.08)	28.0 (3.4–227.6)	2.2 (0.1–34.7)	2.5 (0.2–36.4)
Immobilization	79 (26.4)	19 (1.6)	57.0 (23.0–141.0)	38.9 (14.3–105.6)	28.8 (10.3–80.3)
Infection	124 (41.5)	47 (3.9)	32.4 (18.2–57.5)	25.7 (13.9–47.8)	21.6 (11.5- 40.7)

Abbreviations: CI, confidence interval.
Note: For immobilization, infection, and ischemic stroke, the reference category was defined as no exposure to the trigger.
Model 1: unadjusted odds ratios.
Model 2: adjusted for the other variables in this table.
Model 3: adjusted as in model 2 with addition of major surgery, trauma, red blood cell transfusion, and central venous catheter.
[a]408 deep vein thrombosis cases, four control periods for each case; 299 pulmonary embolism cases, four control periods for each case.

Supplementary Table S5 Distribution of triggers in the hazard and control periods and odds ratios (ORs) of venous thromboembolism after excluding active cancer at the time of the thrombotic event

	Hazard period ($n = 531$) n (%)	Control periods ($n = 2,124$)[a] n (%)	Model 1 OR (95% CI)	Model 2 OR (95% CI)	Model 3 OR (95% CI)
Stroke	27 (5.1)	5 (0.2)	21.6 (8.3–56.1)	8.4 (2.0–34.5)	6.1 (1.5–24.1)
Immobilization	152 (28.6)	37 (1.7)	55.3 (29.1–104.9)	34.5 (17.5–67.9)	24.2 (11.9–49.0)
Infection	166 (31.3)	62 (2.9)	25.3 (16.3–39.2)	16.5 (10.1–26.8)	13.4 (8.1–22.1)
Ischemic stroke	20 (3.8)	2 (0.1)	40.0 (9.3–171.1)	10.2 (1.2–85.4)	8.3 (1.3–54.5)
Immobilization	152 (28.6)	37 (1.7)	55.3 (29.1–104.9)	34.4 (17.5–67.7)	24.3 (12.0–49.4)
Infection	166 (31.3)	62 (2.9)	25.3 (16.3–39.2)	16.6 (10.2–27.0)	13.4 (8.1–22.2)

Abbreviations: CI, confidence interval.
Note: For immobilization, infection, and stroke/ischemic stroke, the reference category was defined as no exposure to the trigger. Stroke includes Ischemic, hemorrhagic, or unclassified stroke.
Model 1: unadjusted odds ratios.
Model 2: adjusted for the other variables in this table.
Model 3: adjusted as in model 2 with addition of major surgery, trauma, red blood cell transfusion, and central venous catheter.
[a]531 cases, four control periods for each case.

References

1 Karlson KB, Holm A. Decomposing primary and secondary effects: a new decomposition method. Res Soc Stratification Mobility 2011;29(02):221–237
2 Breen R, Karlson KB, Holm A. Total, direct, and indirect effects in logit and probit models. Sociol Methods Res 2013;42(02): 164–191
